# Combined Effects of Early Mobilization and Nutrition on ICU-Acquired Weakness

**DOI:** 10.3390/nu17061073

**Published:** 2025-03-19

**Authors:** Paolo Formenti, Alessandro Menozzi, Giovanni Sabbatini, Miriam Gotti, Andrea Galimberti, Giovanni Bruno, Angelo Pezzi, Michele Umbrello

**Affiliations:** 1 SC Anestesia, Rianimazione e Terapia Intensiva, ASST Nord Milan, Ospedale Bassini, 20097 Milan, Italy; giovanni.sabbatini@asst-nordmilano.it (G.S.); miriam.gotti@asst-nordmilano.it (M.G.); andrea.galimberti@asst-nordmilano.it (A.G.); angelo.pezzi@asst-nordmilano.it (A.P.); 2 School of Medicine and Surgery, University of Milano-Bicocca, 20126 Milan, Italy; a.menozzi2@campus.unimib.it; 3 School of Medicine and Surgery, University of Milan, 20121 Milan, Italy; giovanni.bruno@unumi.it; 4 Department of Intensive Care, New Hospital of Legnano, 20025 Legnano, Italy; michele.umbrello@fastwebnet.it

**Keywords:** rehabilitation, nutrition, ICU, ICUAW

## Abstract

Intensive Care Unit-Acquired Weakness (ICUAW) is a very common condition in patients admitted to intensive care units (ICUs), even after relatively short stays. This weakness can develop with a pre-existing background of sarcopenia or cachexia, although these conditions are not always the direct cause. Over the years, much of the literature has focused on the nutritional aspect of the issue, leading to the development of widely accepted guidelines recommending the initiation of early nutrition, with the goal of achieving caloric and protein targets within the first five days of ICU admission. Despite adherence to these guidelines, several studies have shown a significant loss of muscle mass in critically ill patients, which directly impacts their ability to generate strength. However, it has become increasingly evident that nutrition alone is not sufficient to counteract this muscle loss, which is often closely linked to the prolonged immobility experienced by ICU patients due to a variety of clinical and logistical factors. In particular, there is growing evidence suggesting that even the introduction of early and minimal rehabilitation—including passive mobilization—when combined with appropriate nutritional support, can be a valuable strategy to help reduce the incidence of ICUAW. In this narrative review, we aim to summarize the current scientific knowledge on this topic, emphasizing the importance of an integrated approach that combines nutrition and early mobilization. Such a combined strategy not only holds the potential to reduce the acute incidence of ICUAW but also contributes to better recovery outcomes and, eventually, improved quality of life for these patients.

## 1. Introduction

Intensive Care Unit-Acquired Weakness (ICUAW) is a complex syndrome characterized by severe muscle weakness which develops during an ICU patient’s stay and is primarily not attributable to pre-existing conditions [1]. It is closely associated with many conditions, such as prolonged immobilization, systemic inflammation, and the metabolic and neuromuscular disorders associated with critical illness [2]. ICUAW not only complicates functional recovery but also extends ICU and hospital stays, often leading to long-term disability in survivors [3,4]. Even if the loss of muscle mass is a sign of different conditions such as malnutrition, sarcopenia, and cachexia [5,6], recent guidelines emphasize that muscle strength may be a more reliable indicator of adverse outcomes than muscle mass alone [7]. In fact, sarcopenia is characterized not only by muscle loss but also by a decline in muscle quality, with structural alterations at both the microscopic and macroscopic levels [8]. Furthermore, malnutrition accelerates muscle loss and functional decline, which can evolve into physical frailty, leading to impaired mobility and disability. Thus, understanding these interconnected processes help in developing effective interventions aimed to preserve muscle function and improve patient outcomes [9].

In critically ill patients, muscle loss is not simply a consequence of reduced nutrient intake but depends on profound disruptions in metabolic pathways which include dysregulated proteostasis, impaired muscle protein synthesis and breakdown during fasting, disturbances in glucose and insulin homeostasis, inflammation, neuromuscular function, and microvascular function [10]. Moreover, prolonged hospital stays and pharmacological treatments exacerbate long-term complications [11]. Lastly, ICUAW is often a part of Post-Intensive Care Syndrome (PICS), a complex condition that includes physical, cognitive, and psychological impairments that can persist long after ICU discharge [12].

This review explores how early mobilization and nutritional support work synergically to mitigate ICUAW, highlighting their combined role in improving patient outcomes.

## 2. Literature Selection

In this narrative review, we selected relevant literature through a structured search of six major databases: PubMed (1996–present), Embase (1974–present), Scopus (2004–present), SpringerLink (1950–present), Ovid Emcare (1995–present), and Google Scholar (2004–present). We included studies that provided original data or comprehensive analyses on nutritional and rehabilitation support, focusing primarily on randomized controlled trials, observational studies, and meta-analyses. The search strategy involved using keywords such as “early nutrition”, “early rehabilitation”, “muscular assessment”, and “ICU” to identify relevant studies. Two authors, AM and PF, were responsible for retrieving and reviewing the full texts of articles that met the search criteria. They carefully examined titles and abstracts to determine relevance and obtained full-text versions for detailed evaluation. The quality of the selected articles was assessed by evaluating their method, sample size, study design, and relevance to the topic of combined nutrition and rehabilitation in the ICU. Given the nature of a narrative review, we did not perform a formal quality assessment of the included studies, and selection was based on relevance and scientific merit. While this approach allows for a broader discussion of the topic, it also presents limitations compared to systematic reviews, as it may be subject to selection bias and does not provide a quantitative synthesis of the evidence.

## 3. Assessment of Nutritional and Physical Status in ICU

Despite remarkable medical innovations, significant challenges remain in accurately assessing both muscle quantity and quality, making the accurate diagnosis of sarcopenia particularly complex [5]. Determining the nutritional needs of ICU patients adds another difficulty. The traditional measures—such as body mass index, predictive ideal body weight, or adjustments according to body mass index—fail to accurately reflect cellular mass or account for the substantial fluid shifts commonly seen in critically ill patients, leading to potential misinterpretations of nutritional status [13]. Furthermore, serum biomarkers such as albumin, transthyretin, and transferrin, although routinely employed to assess nutrition, also have limitations in critical care [14]. For instance, their levels change due to acute infections, inflammation, and renal or liver dysfunction, making them often unreliable indicators in these settings [15]. Indirect calorimetry has been introduced to overcome these limitations, with the aim of targeting the nutritional needs of each individual patient [16]. The major limitation is that it is not a readily available device in the majority of ICUs [17].

Given these limitations, there is a need for more precise and advanced tools specifically tailored to the physiological complexities of critically ill patients [18]. Nutrition screening should be the first step in this process, integrated into routine patient care. Several validated instruments, including the Malnutrition Universal Screening Tool (MUST) [19], the Nutrition Risk Screening 2002 (NRS-2002) [20], and the NUTRIC score [21,22,23], are commonly used to assess malnutrition risk in ICU patients. Recent efforts have focused on developing numerical screening tools to better identify patients at risk of malnutrition early on. The SCREENIC score was created for this purpose, specifically for intensive care patients [24]. It includes six questions based on patient factors like comorbidities, age, and muscle mass loss, and it was found to have good accuracy in predicting prolonged ICU and hospital stays. One of the most challenging aspects in literature is the lack of a standardized approach to diagnosing ICUAW, without a uniform consensus on the primary diagnostic criteria. Over the years, different studies have progressively integrated complementary diagnostic tools, combining muscle strength assessment with imaging techniques such as ultrasound [25]. In this regard, recently, muscle ultrasound has emerged as a valuable tool for a non-invasive and dynamic method for evaluating both muscle mass and quality. Unlike “static” measurements such as BMI or biochemical markers, ultrasound allows a real-time tracking of muscle changes, providing detailed insights into tissue composition and structure [26]. Parameters such as muscle thickness, echogenicity (which reflects alterations in muscle tissue), and overall architecture can be directly visualized [27]. Despite these highly useful features, it is important to highlight that the ultrasound technique has inherent limitations. These are primarily due to operator-dependent variability in performing the measurement and the specific setting used for muscle assessment, which largely depends on the available ultrasound machine [28]. More recent devices offer dedicated presets that can influence a study’s execution.

Assessing muscle strength is equally important, with various methods available, each with specific applications and limitations. While grip strength correlates moderately with overall limb strength, its utility in ICU patients is often restricted, as critically ill patients may not be able to actively participate in testing during the acute phase of illness [29]. Another widely used approach is the Medical Research Council (MRC) scale, which assesses motor performance on a graded scale from 5 (normal strength) to 0 (no visible muscle contraction) [30]. A prospective cohort study demonstrated agreement between handgrip dynamometry and the MRC score for ICUAW diagnosis [31]. Thus, once patients stabilize and become more cooperative, these assessments offer valuable insights into their functional recovery, guiding rehabilitation strategies with a more personalized approach to patients at a high risk of ICUAW.

## 4. Benefits of Early Mobilization in ICU

Early mobilization in ICU refers to initiating physical activity—including passive movements, active exercises, sitting, or standing—as soon as it is clinically safe for the patient [32]. This practice has gained recognition as a cornerstone of modern critical care due to its substantial impact on the recovery of critically ill patients. Its benefits extend beyond merely preventing muscle loss since early mobilization counteract the physical, the functional, and the psychological consequences of critical illness [33].

### 4.1. Preservation of Muscle Mass and Function

One of the most significant advantages of early mobilization is its role in preserving muscle mass and function [34]. Critically ill patients, especially those undergoing prolonged mechanical ventilation, deep sedation, or extended bed rest, experience rapid muscle atrophy. Research indicates that patients can lose up to 20% of their muscle mass within the first week of immobility [35]. This weakness impacts both peripheral muscles and respiratory function, often leading to difficulties in weaning from mechanical ventilation and prolonged ICU stays [4]. Early mobilization provides a sort of protective strategy against these complications. With this prospective, even apparently minimal movements, such as passive range-of-motion exercises or assisted limb mobilization, play an important role in maintaining neuromuscular integrity [11].

Emerging research is beginning to reveal the molecular mechanisms that explain the positive effects of physical activity on muscle preservation [36]. These processes include molecules like Bassoon, neuregulin-1, and Insulin-like growth factor-1 [37,38].

**Figure 1:**
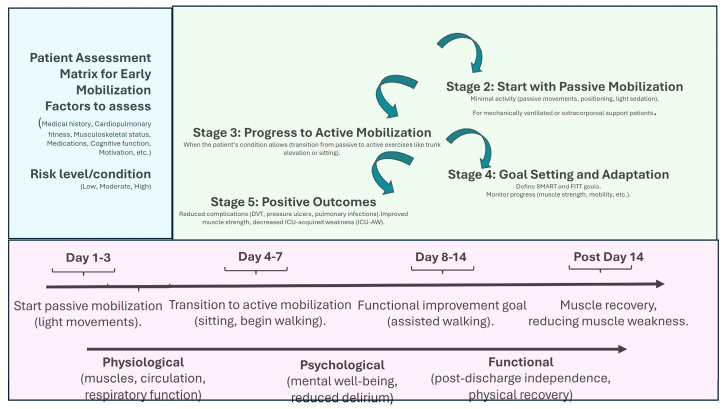
Early mobilization benefits and temporal managing. This figure outlines the step-by-step process for implementing early mobilization in ICU patients. SMART = Specific, Measurable, Achievable, Realistic, and Time-bound; FITT = Frequency, Intensity, Time, and Type.

In terms of rehabilitation, a systematic review by Wang et al. [39], analyzing 60 randomized controlled trials with over 5000 participants, demonstrated that initiating physical rehabilitation in the ICU significantly improved patients’ functional status at hospital discharge and contributed to shorter ICU and hospital stays, even though it did not alter other clinical outcomes. Additionally, Biolo et al. [40] highlighted that exercise improves the muscle’s response to exogenous amino acids, suggesting that movement itself actively contributes to optimizing muscle protein synthesis. Figure 1 shows the main strategies and implications of mobilization programs.

### 4.2. Improved Functional Outcomes

Patients who participate in early mobilization during their ICU stay consistently show better functional recovery than those who remain inactive, and these benefits are particularly evident in post-discharge mobility and self-sufficiency. Research suggests that early mobilization shortens the rehabilitation period post-ICU, accelerating the return to daily activities [41,42]. On the other hand, prolonged immobility in critically ill patients is associated with several complications, including deep vein thrombosis, pulmonary infections, pressure ulcers, and joint stiffness [43]. Early mobilization plays a key role in mitigating these risks since simply sitting up or standing can help expand lung capacity and improve secretion clearance, significantly lowering the likelihood of ventilator-associated pneumonia [44]. A secondary analysis of the PREVENT trial examined how different levels of early mobility during the first three days in the ICU impacted patient outcomes [45]. The findings suggested that patients who were involved in higher levels of mobility had a lower risk of death within 90 days. In addition to these benefits, it alleviated prolonged pressure on vulnerable areas of the body, such as bony prominences, reducing the risk of skin pressure-ulcers and their associated complications [46]. Several studies have consistently reported a direct correlation between early mobilization and improved prognosis in ICUAW. While this evidence strongly supports the beneficial role of early mobilization, some authors have rightly pointed out that multiple factors interact to enhance its effectiveness [47]. Among these are resource staffing, equipment, education, financial support, and the engagement of both staff and family in early mobilization, as well as therapeutic strategies that, in addition to nutrition, include the careful management of sedation and vasoactive drugs, which are directly dependent on the type of underlying pathology.

The ICU environment, often characterized by sensory deprivation, sleep disruption, and frequent sedation, predisposes patients to psychological distress, delirium, and PICS [48]. It has been suggested that mobilization may be able to decrease the incidence of all these detrimental factors [49]. In a randomized controlled trial involving hundreds of ICU patients who had been sedated and on mechanical ventilation for less than 72 h, those who engaged in early mobilization during daily sedation discontinuities were significantly more likely to recover independent functional status by the time of discharge [50].

Despite all these observations, early mobilization also presents potential risks that must be carefully managed. These include the possibility of hemodynamic instability, as physical exertion may lead to variable blood pressure level or heart rate [51]. Furthermore, early mobilization may worsen respiratory function in some patients, particularly those with severe pulmonary conditions [52].

### 4.3. Long-Term Outcomes and Follow-Up Care After ICU Discharge

Many patients who survive a critical illness face long-lasting challenges that can significantly affect their lives. One of the most prevalent concerns is PICS, which encompasses a range of physical, cognitive, and psychological issues that persist well after leaving the ICU [53]. A recent metanalysis comparing early active mobilization with usual care showed that early mobilization was associated with improved physical function in survivors at 6 months [54].

In addition to physical recovery, addressing emotional and cognitive challenges is just as important as physical rehabilitation. In a randomized controlled trial involving 200 mechanically ventilated ICU patients, early physical and occupational therapy (early mobilization) was compared to usual care [55]. The primary outcome, cognitive impairment one year after hospital discharge, was significantly lower in the early mobilization group (24%) compared to the usual care group (43%). The intervention group also showed fewer ICU-acquired weaknesses and better physical quality of life scores. These findings suggest that early mobilization may reduce long-term cognitive impairment but warrant further investigation due to the increased risk of adverse events.

It is also important to provide patients and families with the information and resources they need to understand what to expect in the recovery process and how to manage potential complications [56].

### 4.4. Challenges in Implementation

Despite the well-documented benefits, early mobilization is not without challenges. The most significant limitation is the clinical stability of patients [57]. Mobilization can only proceed when vital signs, hemodynamic parameters, and respiratory function are within safe limits. In the acute phase of critical illness, where mechanical ventilation and sedation are often necessary, active mobilization may not be feasible.

Passive mobilization strategies, such as manual limb movements or passive cycling, can help overcome this challenge, allowing for early intervention while maintaining patient safety [58]. Genc et al. [59] highlighted the positive impact of passive movements in critically ill patients, using a regimen of 10 repetitions for each joint movement. For patients with impaired bowel function, Morisawa et al. [60] showed that passive lower limb and trunk movements, including 10 repetitions of joint movements and an additional 10 min of trunk rotation, were effective. However, other studies have reported mixed outcomes. In a randomized controlled trial including 48 ICU patients, Stiller et al. [61] showed that passive mobilizations, as applied to a cohort of medium- to long-term ICU patients, did not reduced joint stiffness. Therefore, since the evidence on this issue remains inconclusive, it is reasonable to carefully evaluate the appropriateness and timing of initiating passive mobilization on an individual basis, particularly in patients experiencing hemodynamic and respiratory instability.

Another challenge is the need for sufficient staffing and training. Successful mobilization programs require close coordination among intensivists, nurses, physiotherapists, and occupational therapists. This largely depends on the type of organization of the various integrated units [62]. The optimal setting, as suggested in few studies, could involve the implementation of individualized care plans based on principles like SMART (Specific, Measurable, Achievable, Realistic, and Time-bound) [63] or FITT (Frequency, Intensity, Time, and Type). However, implementing individualized care plans is not always straightforward. Some authors have suggested simple key points that could help improve this critical aspect, facilitating a more effective and tailored approach to patient rehabilitation [64,65], in part already discussed above. Regarding mobilization, there are still significant uncertainties about the optimal approach to exercise, particularly in terms of timing, modality, and dosage of interventions. Some evidence suggests that initiating rehabilitation within the first 2–3 days of ICU admission may yield better outcomes compared to later initiation [66]. Various techniques can be employed, including active mobilization, in-bed cycling, neuromuscular electrical stimulation (either alone or combined with passive or active exercises), tilt tables, and different rehabilitation devices. Moreover, factors such as the appropriate intensity, duration, and frequency of these interventions play a crucial role in optimizing their effectiveness [67].

Lastly, a relatively recent development is the introduction of virtual reality in the ICU. Some authors have started to propose the use of these devices in combination with upper- and lower-limb rehabilitation activities. In a proof-of-concept study, virtual reality was shown to be a feasible, safe, and well-received rehabilitation tool [68]. Twenty patients participated in 79 virtual reality sessions using a dedicated app designed for bedridden patients in the supine position. Each session lasted around 14 min, with 10 min dedicated to active training. Importantly, physiotherapists reported no significant barriers, and no adverse events were recorded. Similarly, another author showed a significant impact on mobility scales. A study involving ten ICU patients, all mechanically ventilated for at least 48 h, participated in virtual reality sessions three times a week for 20 min, completing progressively challenging puzzles to improve arm function [69]. Patients completed three weekly sessions, with 13 min of active training per session, and the mobility significantly improved from baseline to the end of the training period [70].

## 5. Benefits of Early Nutrition in ICU

Early initiation of nutrition—ideally within 24–48 h of ICU admission—is recommended in ICU patients [13]. Although guidelines suggest starting nutrition within the first 48 h and reaching the predicted or calculated targets within the first 5 days, there is still debate over whether early initiation should be considered within the first 24 or 48 h, or even beyond this timeframe [71]. In a prospective, randomized trial with 100 ICU patients, those who started enteral nutrition at admission showed significantly higher serum albumin and prealbumin levels, shorter ICU stays and ventilator time, along with fewer complications compared to those who started enteral nutrition at 24–48 h [72]. A meta-analysis including 16 randomized controlled trials found that starting enteral nutrition within 24 h of ICU admission did not reduce mortality compared to other types of nutrition support [73]. However, early enteral nutrition reduced mortality compared to delayed enteral intake.

In any case, the benefits of enteral nutrition have been demonstrated in several studies. Rehal et al. [74] showed how full dosing of enteral nutrition significantly improved whole-body protein balance, which is crucial for improving protein metabolism in ICU patients. However, the optimal timing and approach to nutritional support remain the subject of ongoing debate. For instance, a randomized multi-center trial by Caesar et al. [75] found that delaying parenteral nutrition led to faster recovery and fewer complications, suggesting that the benefits of early full nutritional support may not always be universally applicable and might even cause harm in some cases, as pointed out by Gunst et al. [76]. Moreover, EN was described as being associated with improved mucosal trophism, leading to a reduction in the formation of neutrophil extracellular traps (NETs) and the expression of NET-associated proteins [77]. This effect is linked to the regulation of immune pathways, as early EN attenuated the activation of TLR4, NFκB, and MAPK signaling [78]. On the other hand, providing early nutrition may have some potential risks that need to be carefully considered. Overfeeding can strain metabolic balance, leading to issues like hyperglycemia or liver stress [79], while refeeding syndrome—especially in malnourished patients—can cause dangerous electrolyte shifts and cardiac complications [80]. Digestive issues, such as delayed gastric emptying or diarrhea, may also impact tolerance.

**Figure 2:**
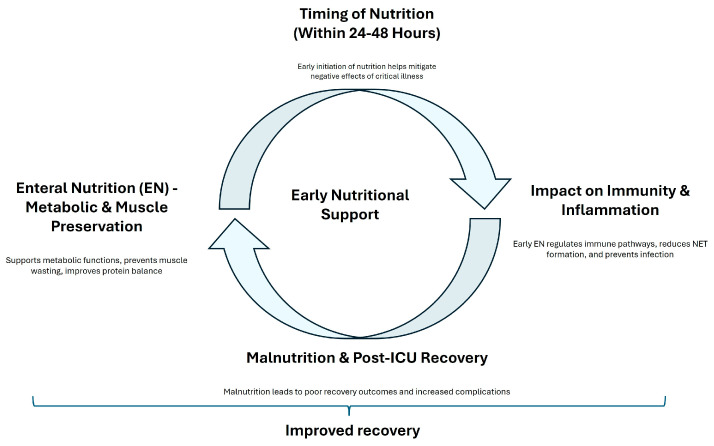
A concept map of early nutritional support. This figure illustrates the concept of ’early nutritional support’ and its impact on patient recovery.

Despite these mixed findings, there is a consensus that nutritional interventions should be personalized based on the individual patients’ needs. Chapple et al. [81] noted that although protein intake often meets international guidelines, the actual delivery of protein to ICU patients frequently falls short. Additionally, Heyland et al. [82] reported that high-dose protein supplementation did not significantly improve hospital discharge times and might even have adverse effects on patients with acute kidney injury. Vallet also demonstrated that negative energy balance is associated with increased complications [83]. The prevalence of malnutrition among ICU survivors, as highlighted by Moisey et al. [84], further supports the importance of ongoing nutritional rehabilitation after discharge. A dual-center randomized controlled trial by Zhou et al. [85] demonstrated that early mobilization combined with guideline-based nutrition significantly reduced the incidence of ICUAW and improved muscle strength compared to standard care. Figure 2 summarized the principal effects of early nutritional support. The first branch emphasizes how EN supports metabolic functions, prevents muscle wasting, and improves protein balance, leading to enhanced recovery. The second branch stresses the importance of early nutritional intervention in mitigating the negative effects of critical illness. A contrasting branch shows the potential benefits of delayed parenteral nutrition, suggesting that postponing parenteral nutrition might reduce complications and accelerate recovery. The third branch focuses on how early EN regulates immune pathways, reduces infection risk, and promotes faster recovery by modulating key signaling pathways. The final branch points out the adverse effects of malnutrition on recovery and the need for ongoing nutritional support after ICU discharge to prevent complications and ensure long-term recovery.

## 6. The Combined Effect of Early Mobilization and Nutrition on ICUAW

The combination of early mobilization and early nutritional support may influence recovery, in essence through muscle preservation and metabolic optimization. Table 1 summarizes the main published observations.

Mobilization stimulates muscle activity, promoting a series of responses that stimulates protein synthesis and improve neuromuscular function [90]. Nakamura et al. [86] showed that high-protein delivery, when paired with active rehabilitation, led to better preservation of muscle volume. Similarly, de Azevedo [87] demonstrated how high-protein intake, alongside resistance exercise, improved physical quality of life and survival rates. Recent studies have highlighted the distinct metabolic effects of exercise and amino acids in regulating anabolic intracellular signaling, muscle protein synthesis, and muscle mass [91]. Mechano-sensors, such as intracellular calcium concentrations and the accumulation of specific molecules, produced by phospholipase D, activate a protein complex involved in muscle synthesis in response to mechanical stress [92]. Nutrient sensing mechanisms, including certain proteins involved in cell signaling, modulate the localization and activation of this protein complex when amino acid concentrations increase [93]. Jones et al. [88] suggested how the combined effects of a 6-week physiotherapy program and an essential amino acid supplement improved patients’ outcomes. They showed that their combination led to improved walking distance, as well as reduced anxiety and depression [71].

Additionally, mobilization activates metabolic pathways related to glucose metabolism and insulin sensitivity. Patel et al. [89] conducted a secondary analysis of 104 mechanically ventilated patients from a randomized controlled trial, comparing early occupational and physical therapy with conventional therapy, focusing on the impact of insulin dose and early mobilization on the incidence ICUAW. Their logistic regression analysis revealed that both early mobilization and higher insulin doses significantly reduced the incidence of ICUAW. A dual-center randomized controlled trial by Zhou et al. [85] demonstrated that early mobilization combined with guideline-based nutrition significantly reduced the incidence of ICUAW and improved muscle strength compared to standard care.

Future studies should focus on adequately powered randomized controlled trials to validate these approaches and further explore the long-term effects of prolonged nutritional interventions on critical patient-centered outcomes such as quality of life and functional recovery [72].

### Practical Implications

**Figure 3:**
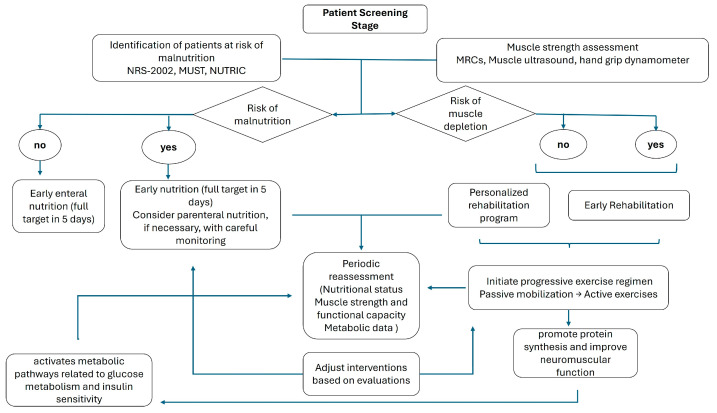
Flow diagram of practical implications.

A detailed flow diagram accompanies a summary of previous observations, delineating the sequential steps—from initial screening and assessment to the implementation and ongoing evaluation of integrated nutrition and rehabilitation protocols (Figure 3).

Initially, rigorous screening procedures must be employed to identify individuals at heightened risk for malnutrition and physical deconditioning. This screening should utilize validated instruments to establish a baseline nutritional profile and determine the need for early intervention. This assessment is particularly important in patients anticipated to have an ICU stay exceeding 72 h, especially in medical patients and those admitted with critical conditions [94]. Conversely, this assessment holds relatively less significance in postoperative patients and those with conditions that are likely to result in a shorter ICU stay.

Following the identification of at-risk patients, a detailed evaluation of muscle strength and function is important. Quantitative assessments, including muscle ultrasound and validated force scales, are recommended to ascertain the degree of deconditioning and to guide the intensity of subsequent rehabilitative measures. Concurrently, it is critical to monitor nutritional intake and energy expenditure accurately. Indirect calorimetry provides a measure of metabolic requirements [76], while serial ultrasound evaluations can yield precise data regarding muscle mass and adipose tissue distribution, thus supplying a comprehensive assessment of the patient’s nutritional and functional status [95].

After these assessments, a patient-specific nutritional plan should be formulated, addressing individualized energy, protein, and micronutrient requirements. Early initiation of enteral nutrition mitigates the risk of further nutritional deficits and supports anabolic processes. In scenarios where enteral feeding is contraindicated or not feasible, parenteral nutrition should be administered with vigilant monitoring of caloric delivery and metabolic parameters [13].

Simultaneously, early mobilization and physical rehabilitation must be planned to counteract the deleterious effects of immobility [96]. A progressive exercise regimen—ranging from passive mobilization to active resistance training—should be tailored to the patient’s current functional capacity. This rehabilitation strategy aims to preserve skeletal muscle integrity, enhance neuromuscular function, and expedite functional recovery.

Periodic re-evaluation of muscle strength and structure with objective measures is essential to refine and adapt the rehabilitation protocol in response to the patient’s evolving clinical status. Similarly, outcome measures should include serial evaluations of nutritional status, muscle strength, functional capacity, and metabolic indices.

## 7. Conclusions

In conclusion, the integrated use of early mobilization and early nutritional support is a fundamental aspect of ICU care that goes beyond addressing muscle weakness or malnutrition. By targeting both the physical and metabolic systems, this combined approach promotes an optimal proposal for recovery, helping critically ill patients regain strength, independence, and overall quality of life after their ICU stay. This synergy between mobilization and nutrition is essential not only for short-term recovery but also for long-term health outcomes, highlighting the importance of integrating these interventions into routine ICU care protocols. Since we did not conduct a formal analysis, as would be carried out in a systematic review, even if the aspects discussed are recognized in other systematic reviews and some guidelines, they should be considered in their specific context.

## Figures and Tables

**Table 1:** Principal investigation on combined effects of nutrition and rehabilitation.

Study	Patients	Design	Main Findings
Hermans et al. [3]	415 ICU patients	cohort study and propensity-matched analysis	ICUAW exacerbates acute health complications, elevates healthcare costs, and is associated with increased mortality rates within one year. The duration and intensity of weakness at the time of ICU discharge are linked to a further rise in one-year mortality rates.
Bragança et al. [31]	45 ICU patients	prospective single-center cohort study	Handgrip strength demonstrated a strong correlation with the MRC criteria for diagnosing ICUAW. ICUAW was linked to an increased duration of mechanical ventilation, extended ICU stays, and longer hospital admissions over a six-month period. No significant differences in mortality rates were observed.
Fazzini et al. [35]	3251 patients	systematic review and meta-analysis	During the initial week of critical illness, patients typically lose about 2% of their muscle mass each day, with continued reductions in muscle mass throughout their time in the ICU. Additionally, approximately 50% of critically ill patients develop ICU-acquired weakness.
Zhou et al. [85]	150 ICU patients	prospective, dual-center, randomized controlled trial	Both early mobilization and early mobilization with nutrition demonstrated beneficial effects. Both interventions may result in a reduced incidence of ICUAW and enhanced functional independence compared to standard care.
Zang et al. [42]	1941 patients	meta-analysis	Early mobilization proved effective in preventing the development of ICUAW, reducing both ICU and hospital lengths of stay, and enhancing functional mobility.
Schweickert et al. [50]	104 ICU patients	randomized controlled trial	A comprehensive rehabilitation strategy led to improved functional outcomes at the time of hospital discharge, a reduced duration of delirium, and an increased number of ventilator-free days in comparison to standard care.
Casaer et al. [75]	4640 ICU patients	randomized multi-center trial (early-initiation vs. late-initiation)	Patients in the late-initiation group experienced a relative increase in the likelihood of being discharged alive. This group also showed a relative decrease of about 10% in the proportion of patients requiring more than two days of mechanical ventilation; the late initiation of parenteral nutrition was associated with a quicker recovery and fewer complications compared to early initiation.
Heyland et al. [82]	1301 ICU patients	multi-center, randomized trial	Administering higher protein doses to mechanically ventilated critically ill patients did not enhance the time to alive discharge from the hospital. A subgroup analysis indicated that increased protein intake was especially detrimental for patients with acute kidney injury and higher baseline organ failure scores.
Nakamura et al. [86]	117 ICU patients	randomized controlled trial	The loss of femoral muscle was significantly lower in the high-protein group compared to the medium-protein group with only active early mobilization.
De Azevedo et al. [87]	181 ICU patients	prospective, randomized controlled trial	The physical component summary was significantly higher in the high-protein and exercise group at both 3 months and 6 months. The control group exhibited markedly higher mortality rates.
Jones et al. [88]	93 ICU patients	randomized controlled trial	Patients who received enhanced physiotherapy, structured exercise, glutamine and an essential amino acid mixture demonstrated the greatest improvements in the 6 min walking test.
Patel et al. [89]	104 patients	secondary analysis of a randomized controlled trial	Logistic regression analyses indicated that early mobilization and higher insulin doses were effective in preventing the occurrence of ICU-acquired weakness, independent of established risk factors for weakness.

## Abbreviations

The following abbreviations are used in this manuscript:

ICU	Intensive Care Unit
ICUAW	Intensive care unit-acquired weakness
PICS	Post-intensive care syndrome
NETs	neutrophil extracellular traps
